# Progress in the treatment of complex osteoporotic proximal humeral fractures in elderly patients

**DOI:** 10.3389/fsurg.2026.1842686

**Published:** 2026-06-23

**Authors:** Wei Fan, Na Yang, Rui Qiao, Kun Zhang, Zhe Song

**Affiliations:** Honghui Hospital, Xi'an Jiaotong University, Xi’an, China

**Keywords:** elderly, intramedullary nail, locking plate, osteoporosis, proximal humeral fracture, reverse total shoulder arthroplasty

## Abstract

Proximal humeral fracture is one of the most prevalent osteoporotic fractures in the elderly, ranking third after hip and distal radius fractures and accounting for approximately 10% of all geriatric fractures. With population aging, its global incidence continues to rise annually. Most mild and stable fractures can be managed conservatively, while nearly 15%–20% of displaced complex fractures require surgical intervention. Current mainstream strategies include locking plate fixation, intramedullary nailing, hemiarthroplasty, anatomic total shoulder arthroplasty, and reverse total shoulder arthroplasty. Owing to poor bone quality and multiple comorbidities in elderly patients, individualized treatment based on fracture classification, medial calcar integrity and functional demands is essential. This review systematically summarizes the clinical application, biomechanical characteristics, functional outcomes and complications of mainstream treatments for elderly proximal humeral fractures. Latest advances in fracture classification, medial calcar reconstruction, biological augmentation, intelligent surgical technology and degradable biomaterials are elaborated. Meanwhile, the limitations of existing studies and future research directions are clarified, aiming to provide evidence-based references for clinical individualized diagnosis and treatment of senile osteoporotic proximal humeral fractures.

## 1 Introduction

Proximal humeral fracture is a common osteoporotic fracture in the elderly, ranking third in overall fracture incidence and occupying about 10% of all fractures in older adults (1, 2). In the context of global population aging, the disease burden of proximal humeral fractures continues to increase. Conservative treatment yields satisfactory long-term functional recovery for most low-displacement fractures, whereas unstable, comminuted and intra-articular fractures are difficult to achieve anatomical reduction and stable fixation through non-operative methods, requiring surgical reconstruction (3).

This article is formulated as a narrative review. Literature was retrieved from PubMed, Embase and Web of Science, focusing on English original articles and reviews published from 2015 to 2025, with search terms including “proximal humeral fracture”, “elderly”, “osteoporosis”, “locking plate”, “intramedullary nail”, “shoulder arthroplasty”. Studies focusing on adult clinical research, biomechanical analysis and material innovation related to proximal humeral fractures were included, while case reports, letters and non-clinical basic studies irrelevant to humeral fractures were excluded.

Elderly patients with proximal humeral fractures are characterized by severe osteoporosis, medial calcar comminution, high complication susceptibility and limited functional tolerance. Fracture classification systems guide therapeutic decision-making, while surgical strategies have gradually shifted from simple internal fixation to minimally invasive reconstruction, medial column support and joint function restoration. This review comprehensively discusses fracture classification, conservative treatment, surgical options and cutting-edge progress, and highlights the imbalance and deficiencies in current clinical research.

## 2 Classification of proximal humeral fractures

### 2.1 Neer classification

Proposed in 1970, the Neer four-part classification (4) divides fractures according to fragment displacement criteria (separation >1 cm or angulation >45°), covering the humeral head, humeral shaft, greater tuberosity and lesser tuberosity. Although the dividing threshold is relatively empirical and arbitrary (5), it remains the most widely used classification in daily orthopedic practice. Nevertheless, the Neer system has obvious defects in evaluating osteoporotic comminuted fractures. Its inter-observer reliability is poor with Kappa values of 0.21–0.48, and intra-observer consistency ranges from 0.45 to 0.64, resulting in low repeatability and persistent clinical controversy (6–11).

### 2.2 AO/OTA classification

The AO/OTA classification optimizes the Neer system by emphasizing fracture morphology and articular blood supply damage. It categorizes proximal humeral fractures into three major types: Type A for simple extra-articular two-part fractures, Type B for double-site comminuted three-part fractures, and Type C for intra-articular or four-part severe fractures. Each type contains detailed subtypes to assist precise preoperative evaluation and prognostic judgment. The 2018 revised AO/OTA classification significantly improves inter-observer consistency and is more suitable for standardized clinical research and complex fracture management (12).

### 2.3 Hertel binary (LEGO) classification

The Hertel LEGO classification divides the proximal humerus into four core anatomical units to assess fracture instability. It focuses on medial calcar integrity, shortening degree and fragment displacement, which are independent predictive indicators of postoperative humeral head avascular necrosis. Severe medial calcar defect and displacement indicate a sharply increased risk of humeral head ischemia, providing an important reference for selecting fixation augmentation or joint replacement (13).

## 3 Clinical treatment strategies

### 3.1 Non-operative conservative treatment

Conservative management is the first-line option for approximately 80%–85% of elderly patients with minimally displaced proximal humeral fractures. Standard protocols include shoulder immobilization with a sling or abduction brace, early pain control, and staged functional rehabilitation training. For older patients with severe underlying diseases, high surgical anesthesia risk and low functional demands, conservative treatment can effectively avoid surgical complications such as nerve injury, implant failure and infection.

Long-term follow-up evidence confirmed that for stable two-part fractures, there is no significant difference in shoulder joint function and quality of life between conservative treatment and surgical fixation (47). The main adverse outcomes of non-operative treatment include shoulder stiffness, mild malunion and chronic pain, which can be largely relieved through standardized early rehabilitation. However, for comminuted four-part fractures, humeral head dislocation and severe medial calcar destruction, conservative treatment often leads to obvious shoulder dysfunction and chronic pain, failing to meet daily activity needs.

### 3.2 Locking plate internal fixation

PHILOS locking plate fixation is currently the most mainstream surgical method for displaced proximal humeral fractures, with continuously increasing clinical application (14). Two common surgical approaches are adopted: the traditional deltopectoral approach and the minimally invasive deltoid-splitting approach. Both approaches can achieve effective fracture reduction, while each has unique advantages and limitations (15).

The deltoid-splitting approach shortens operative time, reduces soft tissue dissection and leaves smaller surgical scars. However, it carries a higher risk of axillary nerve injury and limited exposure of the medial humeral column, making it difficult to reconstruct severe four-part fractures with medial comminution (16, 17). Compared with conventional plates, anatomical locking plates provide superior biomechanical stability, especially for osteoporotic bone. The double-row screw design enhances humeral head holding force; implantation of at least five proximal locking screws combined with medial calcar screws can resist varus stress and maintain fracture reduction (18).

Multiple clinical trials have verified that open reduction and locking plate fixation is superior to conservative treatment in restoring patients' self-care ability and shoulder range of motion (19). However, the overall complication rate of plate fixation reaches 28%–49%, including screw penetration, reduction loss, fracture nonunion and humeral head necrosis. Up to 14%–24% of patients require secondary revision surgery within postoperative follow-up (20–22).

The integrity of the medial calcar is the core determinant of implant mechanical stability. Insufficient medial support is an independent risk factor for locking plate failure. Targeted medial column reconstruction is the key to reducing fixation failure in elderly osteoporotic fractures (23, 24). Current clinical augmentation strategies are summarized as follows:

1) Locking plate combined with medial calcar screws: The inferior medial quadrant of the humeral head has the strongest anti-pullout strength. Screws implanted close to the subchondral bone (about 5 mm below the articular surface) can form effective medial column support and reduce varus collapse (25, 26).

2) Plate fixation combined with bone graft augmentation: Simple artificial bone filling only plays a filling role for bone defects. For large metaphyseal voids caused by osteoporotic compression, structural bone grafts such as allogeneic fibula, femoral head bone and iliac bone are recommended to construct mechanical buttress (27–30). Biomechanical tests have confirmed that structural allograft combined with locking plate significantly improves anti-collapse ability, but high cost, immune rejection risk and potential infection limit its wide application. Long-term studies have shown that for Neer Ⅳ complex fractures, allogeneic bone graft still cannot completely avoid humeral head necrosis and poor functional recovery (31, 32).

3) Dual-plate fixation technique: Lateral locking plate combined with auxiliary medial, anterior or posterior small plate fixation is suitable for fractures with severe medial calcar comminution. Anterior auxiliary plating is the most operable, which protects humeral head blood supply and reduces soft tissue injury; medial double plating provides direct column support but increases neurovascular injury risk; posterior plating prevents posterior tilting but may damage the rotator cuff (33–37).

4) Cement-augmented locking screws: Hollow screws combined with local bone cement injection enhance screw anchorage in osteoporotic bone, reduce screw loosening and cut-out, and effectively lower the early failure rate of internal fixation in elderly patients (38–41).

### 3.3 Intramedullary nail (IMN) fixation

As a central axial fixation technique, intramedullary nailing has unique biomechanical advantages in resisting bending, rotation and shear stress. It is more suitable for fractures with medial calcar comminution that cannot obtain effective lateral plate support (42). In addition, intramedullary nail surgery is minimally invasive, preserves periosteal blood supply, and reduces the incidence of humeral head necrosis.

Early-generation intramedullary nails were limited by unreasonable structural design, accompanied by high rates of rotator cuff injury, acromial impingement and screw loosening (43). Structural optimization of second and third-generation nails greatly improves clinical outcomes: optimized entry point, proximal multi-directional interlocking design and nail-in-nail stable structure effectively reduce mechanical complications (44, 45). Third-generation multi-locking intramedullary nails have achieved reliable fixation effects in elderly osteoporotic fractures (46).

In terms of functional prognosis, current clinical evidence remains controversial. Long-term cohort studies demonstrated that compared with conservative treatment, intramedullary nail fixation cannot bring significant long-term advantages in shoulder function and daily living ability (47). Both locking plate and intramedullary nail fixation can restore basic shoulder function. The selection of fixation methods should be individualized according to fracture classification, bone mass, patient functional expectations and surgeon's technical experience (48).

### 3.4 Shoulder arthroplasty

For elderly patients with severe osteoporosis, four-part comminuted fractures, humeral head splitting or acute dislocation, shoulder arthroplasty is the preferred salvage treatment. Three mainstream prosthetic procedures include hemiarthroplasty, anatomic total shoulder arthroplasty (ATSA) and reverse total shoulder arthroplasty (RTSA).

#### 3.4.1 Hemiarthroplasty & anatomic total shoulder arthroplasty

Hemiarthroplasty and ATSA rely on intact rotator cuff function to maintain shoulder motion. Accurate anatomical reduction and firm fixation of greater/lesser tuberosity are the key to postoperative functional recovery. Suture or wire fixation of tuberosity fragments can promote bone healing; once tuberosity malunion or nonunion occurs, rotator cuff dysfunction and secondary shoulder subluxation will severely affect clinical outcomes (49, 50). These two procedures are mainly indicated for elderly patients with mild osteoporosis, intact rotator cuff and no severe glenoid lesion.

#### 3.4.2 Reverse total shoulder arthroplasty (RTSA)

RTSA changes the shoulder force arm by reversing the glenohumeral joint structure and depends on the deltoid muscle to complete shoulder abduction, which is not limited by rotator cuff injury. Initially designed for rotator cuff tear arthropathy, RTSA is now widely applied in complex proximal humeral fractures in the elderly (51–54).

Patient selection criteria for RTSA: elderly patients over 70 years old, severe osteoporosis, irreconstructable four-part fractures, combined rotator cuff injury, or revision cases after internal fixation failure and hemiarthroplasty malunion.

RTSA can significantly relieve postoperative pain and improve shoulder mobility (55), with stable long-term prosthetic survival. Related data show that the 10-year prosthesis survival rate of RTSA for fracture treatment exceeds 85%, and the main long-term complications include scapular notching and prosthetic loosening. It is inappropriate to simply conclude that ATSA and RTSA have identical efficacy for humeral head avascular necrosis. The supporting literature is limited by small sample size, single-center retrospective design and selection bias, so this conclusion lacks high-level evidence (56). Comprehensive evaluation of glenoid bone mass, rotator cuff condition and activity demand is required for individualized prosthetic selection (57).

## 4 Future development and cutting-edge progress

Current surgical treatment of elderly proximal humeral fractures still faces bottlenecks such as high internal fixation failure rate in osteoporotic bone, difficult medial calcar reconstruction and limited long-term joint function recovery. With the development of tissue engineering, intelligent medicine and degradable biomaterials, the treatment of proximal humeral fractures is developing toward minimal invasiveness, precise individualization and functional regeneration. All contents in this section are closely combined with the research status of proximal humeral fractures, avoiding irrelevant extrapolation from other skeletal systems.

### 4.1 Biological augmentation for osteoporotic fracture healing

#### 4.1.1 Stem cells and 3D-printed scaffold technology

Bone marrow mesenchymal stem cells (BMSCs) combined with 3D-printed porous scaffolds have shown good application potential in the repair of osteoporotic bone defects (58). For compressed bone defects and metaphyseal bone loss after proximal humeral fractures, 3D-printed composite scaffolds loaded with osteogenic factors and stem cells can simulate bone microstructure, promote local angiogenesis and osteogenic differentiation, and improve bone mass around implants. Existing preclinical studies on osteoporotic fracture models have verified that this technology can accelerate fracture healing and enhance local bone strength, which is expected to solve the problem of poor bone healing in elderly patients (59–61).

#### 4.1.2 Concentrated platelet-rich fibrin

Concentrated platelet-rich fibrin (C-PRF) contains abundant platelets, leukocytes and growth factors, which can be locally implanted during surgery to promote soft tissue repair and bone regeneration. In humeral fracture surgery, local application of C-PRF can reduce postoperative inflammatory reaction, accelerate tuberosity bone healing, and lower the risk of delayed union after internal fixation or arthroplasty (62).

### 4.2 Intelligent and precision surgical technology

#### 4.2.1 Orthopedic robotic surgery

At present, joint replacement robots have been maturely used in hip and knee arthroplasty, while fracture reduction robots are mainly applied in pelvic and femoral fractures (63–65). Although robotic-assisted minimally invasive surgery has not been widely popularized in proximal humeral fractures, several center trials are exploring robotic-assisted precise reduction and screw placement for PHF. Robots can realize preoperative 3D fracture reconstruction, intraoperative real-time navigation and accurate screw implantation, effectively reducing the risk of neurovascular injury and screw penetration, and improving the accuracy of medial calcar reconstruction (66, 67).

#### 4.2.2 AI-Assisted preoperative planning

Artificial intelligence and big data technology provide new tools for individualized treatment of proximal humeral fractures. Based on preoperative CT data, AI can automatically reconstruct 3D fracture models, simulate different internal fixation schemes and prosthetic matching effects, and predict the risk of postoperative collapse and complications. This data-driven decision-making model reduces the deviation caused by personal surgical experience and provides standardized preoperative planning for complex elderly fractures (68–70).

### 4.3 Degradable magnesium alloy internal fixation materials

Magnesium alloy has biomechanical properties close to human cortical bone, good biocompatibility and controllable degradation performance, which is an ideal new generation of orthopedic internal fixation materials. Degradable magnesium alloy locking screws and mini-plates for proximal humeral fractures can avoid secondary removal surgery. Through surface coating modification, the degradation rate of magnesium alloy matches the fracture healing cycle, which can provide early stable fixation and reduce long-term implant foreign body reaction. At present, related animal experiments and small-sample clinical trials have achieved preliminary good results in osteoporotic fracture repair (71, 72).

## 5 Limitations

This study is a narrative review without strict systematic retrieval and meta-analysis. The included literature quality is uneven, and most clinical studies are single-center retrospective trials with inconsistent evaluation indicators. The efficacy comparison of different surgical methods lacks unified quantitative data of Constant score, ASES score and long-term revision rate. In addition, some emerging biological materials and intelligent surgical technologies are still in the preliminary exploration stage for proximal humeral fractures, and large-sample, multi-center prospective studies are still needed to verify their safety and clinical promotion value.

## 6 Conclusion

The treatment of elderly complex osteoporotic proximal humeral fractures requires hierarchical management based on fracture classification, medial calcar integrity, bone quality and individual functional demands. Conservative treatment is the preferred regimen for stable minimally displaced fractures; locking plate fixation combined with medial column reconstruction is the mainstream for most comminuted displaced fractures; intramedullary nailing has unique minimally invasive advantages; and reverse total shoulder arthroplasty is the optimal choice for severe complex fractures and rotator cuff insufficiency.

In the future, combined with biological osteogenesis enhancement, artificial intelligence navigation surgery and degradable biomaterials, the clinical management of proximal humeral fractures will further realize precise minimally invasive treatment and long-term functional reconstruction. Standardized multi-center clinical research and unified functional evaluation criteria should be established to provide more high-level evidence for the optimization of treatment strategies for elderly proximal humeral fractures.
